# Influence of psychosocial safety climate on occupational health and safety: a scoping review

**DOI:** 10.1186/s12889-023-16246-x

**Published:** 2023-07-13

**Authors:** Mustapha Amoadu, Edward Wilson Ansah, Jacob Owusu Sarfo

**Affiliations:** Department of Health, Physical Education and Recreation, University of Cape Coast, Cape Coast, Ghana

**Keywords:** PSC, Job demand, Job resources, Occupational health and safety, Scoping review

## Abstract

**Background:**

Creating a healthy, decent and safe workplace and designing quality jobs are ways to eliminate precarious work in organisations and industries. This review aimed at mapping evidence on how psychosocial safety climate (PSC) influence health, safety and performance of workers.

**Methods:**

A literature search was conducted in four main databases (PubMed, Scopus, Central and Web of Science) and other online sources like Google Scholar. A reference list of eligible studies was also checked for additional papers. Only full-text peer-reviewed papers published in English were eligible for this review.

**Results:**

A search in the databases produced 13,711 records, and through a rigorous screening process, 93 papers were included in this review. PSC is found to directly affect job demands, job insecurity, effort-reward imbalance, work-family conflict, job resources, job control and quality leadership. Moreover, PSC directly affects social relations at work, including workplace abuse, violence, discrimination and harassment. Again, PSC has a direct effect on health, safety and performance outcomes because it moderates the impact of excessive job demands on workers’ health and safety. Finally, PSC boosts job resources’ effect on improving workers’ well-being, safety and performance.

**Conclusion:**

Managers’ efforts directed towards designing quality jobs, prioritising the well-being of workers, and fostering a bottom-up communication through robust organisational policies, practices, and procedures may help create a high organisational PSC that, in turn, promotes a healthy and decent work environment.

**Supplementary Information:**

The online version contains supplementary material available at 10.1186/s12889-023-16246-x.

## Introduction

Every job has tremendous inherent health, safety and well-being challenges, thus, creating a safe and decent work for improved health and safety outcomes becomes eminent [1, 2]. For instance, pprecarious jobs and work environment are detrimental to the health and safety of workers and place huge financial burden on workers and their organisations [2]. Occupational incidents do not affect only workers and their families, but have a huge burden on society through impaired productivity and increased use of healthcare and cost [3, 4]. According to the International Labour Organisation (ILO) working conditions are worsening globally, and majority of workers are found in precarious employment [1], which is responsible for about 7,600 deaths daily [1]. Therefore, occupational health and safety (OHS) remains the key factor to restoring dignity at work and improving worker health outcomes, to meet the Sustainable Development Goal (SDG) target 8, which seeks to eliminate all forms of precarious work and ensure a decent and safe workplace for all [1]. However, robust research designs and reviews are needed to map quality evidence to inform interventions and policies aimed at creating such a safe and decent work for all workers.

Evidence from the World Health Organisation (WHO) and ILO shows that in 2016, about 1.9 million deaths occurred globally due to occupational accidents and injuries [5]. Again in 2017, about 2.78 million workers died from occupational-related accidents and injuries [6, 7]. Thus, globally, about 7,600 workers died daily in 2017 due to precarious and unhealthy working conditions, but this affects poor developing nations disproportionately. For instance, the African region recorded the highest global occupational communicable diseases among over one-third of its working population and 20% of its workforce has experienced serious work-related accidents [1]. These unfortunate trends of statistics are frightening and might be as a result of insufficient safety regulations and enforcement as well as emerging industries and technological advancements which may require updated safety protocols and training [1]. Also, these figures give the indication that most workers, especially those in developing countries do not have access to a decent, safe and healthy workplace [5, 8]. Perhaps, global economic pressures are forcing some industries and organisations to focus on cost-cutting and increase productivity instead of protecting the well-being and safety of their workers [1]. There is the need for adequate measures and pragmatic steps taken by national regional and global bodies to guarantee decent, safe, and healthy workplace for all workers [5, 8]

Evidence shows that global occupational morbidity and mortality from psychosocial hazards keep increasing, something that need urgent attention [5, 8]. Psychosocial working conditions or exposure to psychosocial hazards by workers, to a greater extent, is dependent on the interplay between job demands and job resources (job design) [9, 10]. Most work stress models such as the job demand-resource, job demand-control and effort-reward imbalance argue that work environments with high job demands and fewer job resources expose workers to impaired health outcomes that lead to impaired performance and less productivity [11]. Psychosocial safety climate (PSC) has been the basis for job designs and improving social relations at work, perhaps it is capable of prioritising the well-being and safety of workers [12]. Besides, PSC is capable of buffering the effect of high job demands on workers’ health and safety [11].

In organisations with high PSC, the well-being and safety of workers are prioritised [11, 12], commitments and efforts are made by senior management to involve and leverage workers’ participation in designing jobs and programmes that help create a safe and healthy work environment for improved well-being, safety and productivity [12]. Empirical evidence from work stress, organisational psychology and safety science showed PSC as a unifying framework for dealing with work stress [11]. While there is a growing body of research work exploring PSC, not enough is understood about its importance and application to psychosocial working conditions, health and safety, and performance of workers. Hence, this review maps evidence on the influence of PSC on psychosocial working conditions, health and safety, and performance, thus, to inform workplace policies and actions that create a safe, decent and healthy workplace for all workers to achieve SDG 8 and improve organisational performance.

## Methods

The authors carried out this scoping review using the guidelines by Arksey and O’Malley [13], by identifying and stating the research questions, identifying relevant studies, study selection, data collection, data summary and synthesis of results, and consultation. Two research questions guided this review. (1) What is the influence of PSC on (a) psychosocial work factors, (b) health and safety outcomes of workers, and (c) performance and productivity outcomes? (2) what is the moderating role of PSC in the health erosion and motivation pathways?

Authors created a search technique that employed a combination of controlled vocabularies like Medical Subject Headings (MeSH) and keywords for each of the four major electronic databases (PubMed, Scopus, Central and Web of Science) to address the research questions and map relevant literature. Table 1 illustrates the search strategy conducted in PubMed. The search strategy used in PubMed was then modified for search in other databases. The authors used four key words in their search strategy (1) psychosocial safety climate, (2) psychosocial work factors, (3) Health and safety and (4) performance.

**Table 1:** Search strategy used in PubMed to retrieve scientific literature

*#*1	It identifies safety climate	Psychosocial safety climate*[MeSH Terms] OR psychological safety climate* OR and psychosocial safety culture* OR safety climate*
*#2*	Search to identify psychosocial hazards	Psychosocial work factors* [MeSH Terms] OR psychosocial working conditions* OR lone working* OR workplace bullying* OR workplace abuse* OR job demands* OR psychological job demand* OR emotional job demands* OR physical work demands* OR job stress* OR job resources* OR job control* OR job autonomy* OR skill discretion* OR organisational justice * OR organisational leadership* OR work-family conflict* OR social support* OR co-worker support* OR supervisor support* OR organisational support* OR workplace harassment* OR Job-related stress*
*#*3	Search to identify health and safety outcomes	Health and safety* [MeSH Terms] OR psychological well-being* OR quality of work life* OR quality of life* OR injuries* occupational diseases* OR exhaustion* OR fatigue* OR burnout* OR emotional burnout* OR morbidity* OR mortality* compensation claims* OR insurance claims* OR stress* OR depression* OR anxiety* OR mindfulness* OR death* OR illness* OR mental distress* OR mental health* OR circulatory diseases* OR fatality* OR kidney diseases* OR diseases
*#*4	Search to identify performance outcomes	Performance* [MeSH Terms] OR productivity* OR work engagement* OR job satisfaction* OR customer satisfaction* OR patient safety* OR personal growth* OR organisational commitment* OR profit* OR workaholism* OR innovative behaviours* OR absenteeism* OR presenteeism* OR job satisfaction* OR safety participation* OR safety behaviours*
	Overall search strategy	#1* AND *#*2 AND *#*3AND *#*4 NOT animal* **(Filters activated: English, from 2010/01/01)**

Additional searches were conducted in Google, Google Scholar, JSTOR, Emerald, and Taylor and Francis to gather adequate and relevant peer-reviewed papers for this review. Reference lists of eligible full-text articles were also searched for additional papers. A chartered librarian was consulted during the search for literature and data screening process. The authors started the search for papers on December 5, 2022, and ended on March 29, 2023. The authors developed eligibility criteria for data screening. Studies published in the year 2010 and later were included because we were interested in studies that explored PSC using PSC-12 and that PSC-12 was published in 2010 [12] (See Table 2 for details on eligibility criteria).

**Table 2:** Eligibility criteria for screening search results and full-text records

**Inclusion criteria:**
1. The paper is written or published in the English language; 2. Only peer-reviewed articles; 3. The study should explore the psychosocial safety climate among the working population; 4. The study adopted or adapted the PSC-12, PSC-8 or PSC-4 to measure psychosocial safety climate or interview participants; 5. The study was conducted in any part of the world; 6. The study was published online in the year 2010 or later
**Exclusion criteria:**
1. The paper was written or published in any other language other than English; 2. The paper is a conference paper, a letter to the editor, pre-print, grey literature, and commentaries; 3. The paper did not explore psychosocial safety climate but related constructs such as safety climate, physical safety climate, safety culture, etc.; 4. The paper did not adopt or adapt PSC-12, PSC-8 or PSC-4 in measuring psychosocial safety climate; 5. The paper was published before the year 2010 (Psychosocial safety climate was introduced in the year 2010); 6. Abstracts without full-text records; 7. The study was published online before the year 2010

The Mendeley software was used to remove duplicates. Abstracts and full-text records were screened and papers selected based on eligibility criteria. Data from eligible papers were extracted independently by MA and reviewed by EWA and JOS. Disagreements among authors during the data screening and extraction phases were resolved during weekly meetings to ensure accuracy in extracted data. Data extracted included authors, purpose of the study, design, population, sample size, measure for PSC, and study outcomes. These data were relevant to help map evidence to answer the research questions and make relevant recommendations for future studies. Extracted data is presented in Table S1. The authors read through the final extracted data, organised data into themes and results presented and discussed.

## Results

### Search results

**Fig. 1:**
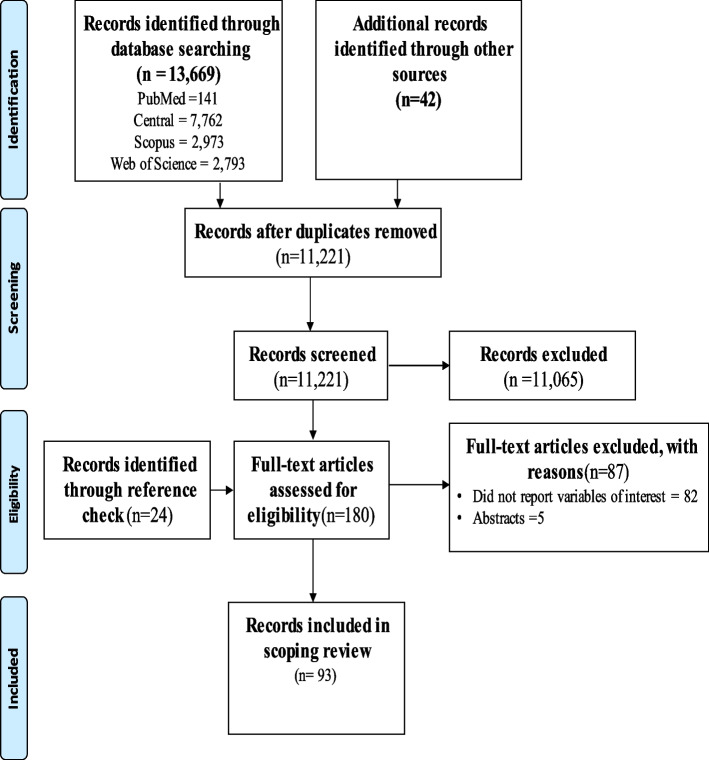
PRISMA flow diagram of search results and screening process

The results from the four main databases yielded 13,669 records and additional search produced 42 records. After removing duplicates (2,490 records) using the Mendeley software, 11,221 records were available for screening. After removing non-full text and records irrelevant to the review, 156 full-text records were available for further screening. Checking of reference lists of full-text records produced additional 24 records. Thus, 180 records were finally screened. Finally, 87 full-text records were excluded, the remaining 93 were included in the thematic synthesis (See Fig. 1 for search results and screening process).

### Study characteristics

**Fig. 2:**
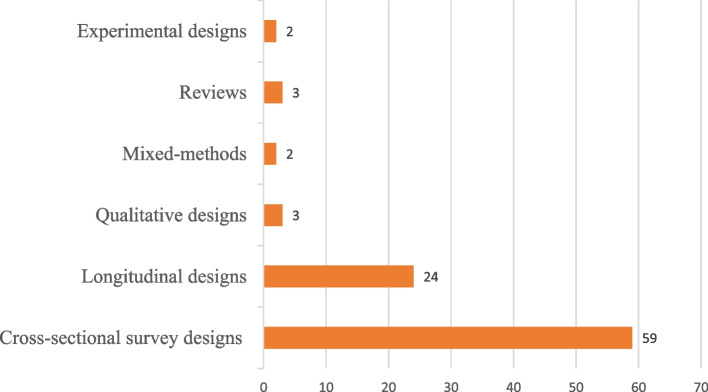
Study designs of reviewed studies

**Fig. 3:**
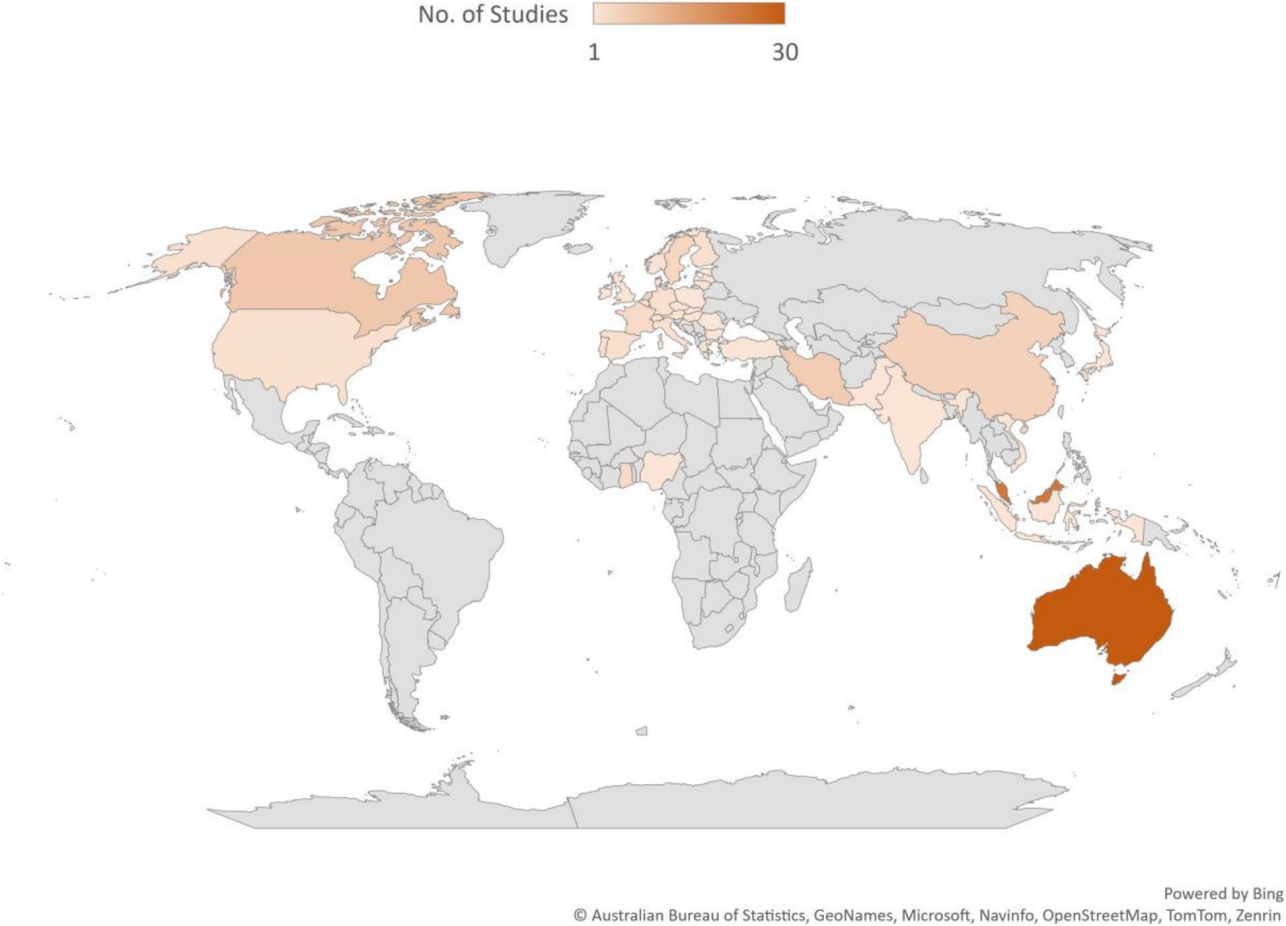
Map showing countries and continents where reviewed studies were conducted

**Fig. 4:**
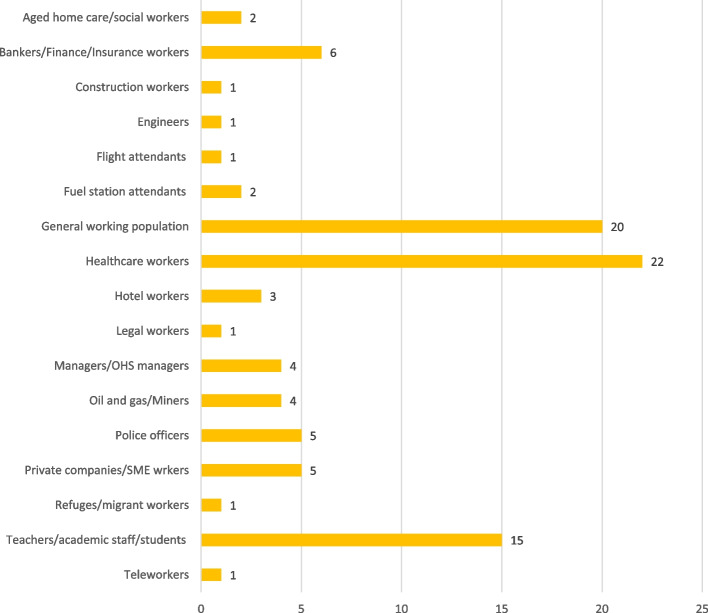
Occupational groups explored by reviewed studies

**Fig. 5:**
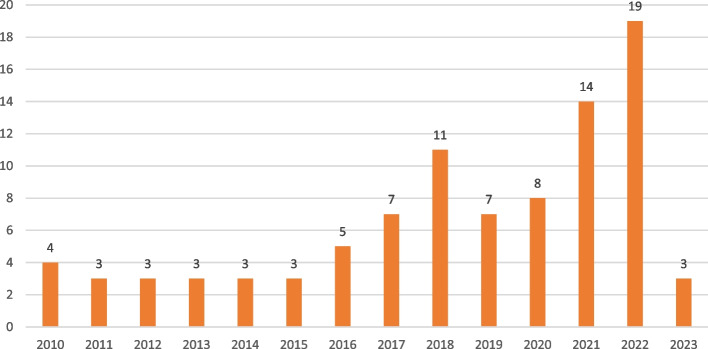
Number of studies based on the year of publication

Most reviewed studies used a cross-sectional survey design (See details in Fig. 2), and were conducted among workers in Australia (30) and Malaysia (24) [See details in Fig. 3]. The general working population, healthcare workers and workers in academia remained the most explored groups using PSC (See Fig. 4 for more details). Most of the reviewed studies were published in the year 2022 (See Fig. 5 for more details).

### Findings

Findings from this review were reported based on the two research questions, and into four sections; (1) influence PSC on psychosocial work factors, (2) influence of PSC on health and safety, (3) influence of PSC on performance outcomes and (4) the moderating effect of PSC.

### Influence of PSC on psychosocial work factors

Three sub-themes were developed from the findings of the reviewed studies. The themes are job demands, job resources, and hostile work factors.

#### Job demands

Evidence is strongly established in the literature that PSC is negatively and significantly associated with job demands [12, 14–19]. PSC has a significant and negative association with cognitive demands [20], psychological demands [15, 21–23], emotional demands [22, 24–26], quantitative demands [27], work intensification [28], work pressure [25, 29], conflicting pressure [30], workload [25], long-working hours [31], hindrance demands [32–36], challenge demands [32] and compulsive working [37]. However, a reviewed study found no significant association between PSC and challenge hindrance [36]. Job insecurity [38], work-family conflict [14, 38–40], effort-reward imbalance [41] and family-work conflict [39] are reduced in high PSC context.

#### Job resources

Job resources are high in a positive PSC context at various occupational settings [17–19, 21, 42–45]. Key job resources such as job control [26, 46, 47], decision authority [21], decision influence [48], skill discretion [21, 25], co-worker support [38], supervisor support [22, 46], managerial support [49], organisational support [50] and organisational rewards [22, 51] were found to have a positive and significant association with PSC. Furthermore, workers in a high PSC work environment were more likely to perceive a high possibility for development [20], organisational justice [22, 52], health-centric [53], quality leadership [27, 54], psychological capital [55] and emotional resources [45] at work.

#### Workplace abuse

PSC had a negative and significant association with workplace bullying [29, 51, 56–61], and that, workplace violence [29, 62], physical or verbal abuse [63], and harassment [29, 51] were reduced or eliminated in the presence of a high-level PSC.

### Influence of PSC on worker health and safety

Findings indicated that burnout [19, 27, 33–35, 59, 64], job strain [65, 66] and emotional exhaustion [21, 22, 24, 25, 48, 61, 67] might be a result of low organisational PSC. Also, fatigue [68, 69], injuries [67], accidents [70] and circulatory diseases [71] had a significant and negative association with PSC. Moreover, mental health issues such as psychological distress [23, 26, 42, 49, 54, 64, 67], stress [27, 72], depression [31, 41, 65, 73] and PTSD [56] might be a result of low workplace PSC. Meanwhile, reviewed studies found that workers that perceived high levels of PSC at work were more likely to experience improved general health, safety and well-being [12, 16, 17, 55, 57, 62, 74], psychological well-being [15, 58], personal resilience [75], psychological safety [54, 76], and self-worth [77].

### Influence of PSC on job performance outcomes

Improved job performance was linked to higher perceived organisational PSC [32]. Similarly, job satisfaction [17, 27, 77, 78], work engagement [17, 21, 22, 25, 27, 37, 42, 57, 69, 79, 80] and job commitment [27, 44, 52] are three key performance outcomes (psychosocial outcomes) that were consistently reported to be associated with high level of PSC. However, two studies reported no significant association between PSC and job engagement [44, 81], but improved productivity was expected in a highly perceived PSC work environment [20, 75]. As a result, issues that affected productivity, such as turnover intentions [41, 61, 78], absenteeism [71, 82, 83], presenteeism [23, 28, 82, 84], and need thwarting [40] were reduced or eliminated in highly perceived PSC work environment. These might lead to more funding opportunities [47], sustained profits [83] and reduced compensation claims [83].

Elimination of unsafe working behaviours [85] and improvement in workplace safety behaviours [38, 86], safety participation [87] and compliance [87] were also common in workplaces where management prioritises the well-being of workers. Workers were more likely to be workaholics [44], have high morale [83], and develop organisational citizenship behaviours [50] in a high PSC context. Moreover, adaptive and proactive work behaviours [88], creative problem solving [55, 89], taking of personal initiatives [80], personal development [80], positive service behaviour [88], workaround [68], and service recovery performance [90] were more likely to be observed in high PSC work environment. Perhaps, managerial quality is one of the key benefits in a high organisational PSC context [64, 91]. For instance, the quality of patient care and patient safety was protected when healthcare professionals perceived high PSC in their facilities [30, 70].

### The moderating role of PSC

One key strength of PSC was its buffering effect on precarious work conditions on health, safety and performance outcomes [11].

#### The effect of workplace abuse on workers’ health and safety

The effect of workplace abuse and violence on workers’ health and safety is controlled by the presence of PSC. For instance, reviewed studies reported that PSC moderated the effect of workplace bullying on psychological contract violation [92], work engagement [57, 79], PTSD [56]and psychological distress [52]. Also, PSC played a moderating role in the effect of workplace harassment on psychological distress [52], and the impact of workplace stigma on bullying and burnout [59]. Contrary to the argument of Dollard et al. [11], the moderating role of PSC on the association between workplace bullying and psychological contract violation had an inverse result [92].

#### The effect of job demands on workers’ health and safety

Evidence also indicated that PSC could buffer the effect of job demands on workers’ health and safety. For example, the effect of job demands on burnout [81], fatigue [69], work engagement [69] and depression [93] were found to be moderated by PSC. Also, the association between emotional demands and emotional exhaustion [12] and psychological distress [94] were reduced in the presence of high-level organisational PSC. Furthermore, the relationship between work-family conflict and insecurity, as well as the association between job insecurity and safety behaviours are buffered by the presence of workplace PSC [38]. The high level of workplace PSC among nurses reduced the effect of work intensity on presenteeism [29].

#### The effect of job resources on workers’ health and safety

It was expected that in a high PSC work environment, job resources’ effect on workers’ health and safety would be enhanced [11]. For instance, the effect of job resources on safety behaviours [38], and workaholism [43] were boosted in the presence of high PSC. Evidence also showed that the effect of social support (support from co-workers and supervisors) on work engagement [81]and the effect of job control on mindfulness among workers improved in the presence of high PSC [95]. Moreover, a reviewed study found that a supportive work environment’s effects on personal hope were lowered in low PSC [76]. Besides, health-centred leadership had the greatest impact on psychological health when oil and gas workers perceived high PSC [53]. Finally, the interaction between job demands and job resources in predicting distress among police workers was moderated by PSC [26].

#### The effect of mental health on workers’ behaviours

In an unsafe work environment, workers’ mental health is severely impaired [88]. In such a situation, the high presence of PSC is expected to control the effect of mentally distressed on workers’ performance [12]. A reviewed study confirmed this hypothesis and reported that the effect of depression on workers’ positive organisational behaviour was attenuated by the presence of PSC [17].

## Discussion

A thorough literature search conducted in PubMed, Scopus, Central and Web of Science and other databases such as Google and Google scholar produced 13,669 records. Through a robust screening process, 91 studies that explored psychosocial safety climate using PSC-12, PSC-8 and PSC-4 as a measure were included in this review. Reviewed studies showed that PSC, as an upstream job resource construct, was essential in designing jobs by matching job demands and resources. Thus, PSC has consistently been found in the literature to be negatively associated with job demand variables such as psychological demands, emotional demands, quantitative demands, work intensification, work pressure, conflicting pressures, job insecurity, work-family conflict, family-work conflict, and effort-reward imbalance. Moreover, PSC is positively associated with job resources (job control, social support, quality leadership, organisational rewards, decision authority and influence, emotional resources, organisational justice, and personal development). Hence, PSC has great influence on psychosocial work factors (job demands and job resources). Also, it was established that PSC was negatively associated with workplace abuse, such as stigma, discrimination, bullying, and harassment. Furthermore, PSC directly improves workers’ health, safety, and performance, proving a strong buffering effect for health and safety of workers. This shows that PSC has influence of health and safety and performance outcome of workers and reduce the effect of precarious work on the health and safety of workers. Discussion of findings have been done according to the research questions.

### PSC as a precursor to psychosocial work factors (job demands and resources)

Managers need to be guided by ethics and value for workers when making decisions regarding job design and nature to foster healthy and decent workplaces [96]. However, job design and the promotion of a healthy and decent workplace might depend on the priority managers give to productivity or profits as against the well-being and safety of the workers [11]. In many cases where the manager’s priority was overly focused on productivity and profits, job demands were high, affecting workers’ health and safety, especially in a resource-limited work environment [12]. However, when managers shift attention from productivity to well-being and safety of their workers, excessive job demands are likely to be reduced, to protect the health of the workers. Perhaps, the negative association between job demands and PSC is explained by the shift of managers’ attention from productivity to valuing the psychological well-being and safety of the workers and vice versa.

The review further found that in a low PSC context, excessive job demands are expected, due to the lack of feedback from workers or the lack of opportunity for workers to voice their frustrations concerning high level of job demands [59]. In such organisations, job demands were likely to be high because of the likelihood that managers prioritised an up-to-bottom communication rather than a bottom-up approach, to ensure that workers’ voices are heard and factored into the job designs [43, 95]. There is high likelihood of reduced job demands when organisational PSC is high, because workers will be involved, consulted, participated in designing their jobs, workplace health and safety policies and any intervention that creates a healthy and decent workplace for such workforce. Finally, PSC was observed as an upstream job resource and its presence at the workplace is a signal for reduction in excessive job demands and helping workers to fulfil their requirements, that achieve organisational goals and a sense of belongingness [11].

The quality of a worker’s productivity or performance is influenced by the design of their job, which also establishes how workers would carry out their responsibilities and meet organisational and personal goals. It is worth appreciating that quality work involved resourcing workers adequately to cope with excessive job demands [12, 44]. The positive association between job resources and PSC indicates that in a high PSC work environment, workers have the confidence to access the needed resources to accomplish their job demands and responsibilities [43]. Thus, in such a context, workers are encouraged, trained and offered the opportunities not only to access job resources but to utilise these resources for organisational and personal growth [11]. Besides, in a high PSC context, adequate job resources are made available to workers to ensure that the psychological well-being of workers are prioritised over productivity. On the other hand, in a low PSC context, job resources were limited and, to a larger extent, non-existing, which exposes workers to job strain and poor health outcomes [12], that will further compromise productivity.

Workplace abuse and violence are unhealthy factors that exposed workers to precarious situations. We found that workplace abuse, bullying, harassment, stigma and discrimination were social-relational factors that created an unhealthy, corrupt and indecent workplace, violated human rights, and compromised the dignity of workers [56, 61]. Various mechanisms might explain the negative association between PSC and workplace abuse. First, in a high PSC context, workplace social relations are supposed to improve and give workers the signal that there are available resources for dealing with any form of abuse [12]. Also, workers who are abused victims were given opportunities to find solutions in such positive worksites [12, 71]. This way of solving workplace conflicts or abuse might not be present in a low PSC work context which may fuel turnover intentions and turnovers of affected workers [41, 78]. Finally, it would be difficult for many workers to report abuse in organisations where PSC is low, and that majority of these workers may not have the opportunity to seek redress since such institutions practice the top–bottom approach communication that usually limits open communication and trust in management [41]. But, in a high PSC context, managers give cues to workers about social-relational aspects of work, such as how workers should interact with one another and the behaviours that would be rewarded or punished [12].

### PSC as a precursor to workers’ health and safety

Evidence suggests that PSC positively correlated with improved worker health, safety and performance outcomes [13, 97]. High-quality work with manageable job demands, and adequate job resources were more likely in a high PSC work environment, where managers value and safeguard workers’ psychological health for improved well-being, safety and performance outcomes [18, 59, 66]. Thus, a high PSC context foster satisfaction of psychological needs, job satisfaction, job commitment, and mental health maintenance, which translate into improved productivity [58, 88, 89]. Basically, in such a PSC context, workers perceive that their well-being is a priority to managers, hence, become intrinsically motivated, which may lead to improved mental health and well-being [98, 99], and positive performance outcomes. Unfortunately, low PSC environments are more likely to produce low-quality work that threatens and obstructs worker job satisfaction, resulting in psychological distress, exhaustion, fatigue, impaired well-being and organisational performance [62, 68].

### The moderating role of PSC

The evidence is that PSC moderates the effect of psychosocial work factors on health, safety, and performance outcomes [26]. One explanation is that PSC acts as a safety signal [52], when danger cues such as work pressure, excessive job demands, and workplace abuse are present. This safety signal works by indicating options such as access to and safe use of available resources to counteract the psychosocial hazards to prevent the onset of impaired health, safety and performance outcomes [26, 54]. Aside from being a safety signal, PSC could initiate resource caravans or gain spirals, promoting workers’ well-being and productivity [96]. A study found that PSC moderated the association between workplace bullying and psychological contract violation [93]. It is worth noting that receiving support at the workplace was not always be connected with favourable health and performance outcomes, primarily when the support is obtained in an unsafe or negative work environment [93], making the organisational climate increasingly important.

### Implications for practice

Creating and promoting a healthy, safe and decent workplace might start with integrating PSC as an essential upstream psychosocial resource at every workplace. Moreover, efforts directed towards prioritising and valuing the well-being and safety of workers by managers may be the beginning of eliminating precarious working condition. Thus, the experience of workers at the workplace, to a greater extent, influence workers perception of PSC. Still, this premise does not change the fact that managers possess the power and resources to design quality jobs through pro-worker and robust organisational policies and practices [12]. Dollard et al. [12] argued that PSC was a modifiable variable; hence, managers should know that change could be implemented by improving involvement and communication mechanisms around psychosocial hazards and mental health issues. This could also be achieved by management demonstrating commitment and support for stress prevention and psychological treatment. Furthermore, managers commitment becomes paramount to any workplace policies targeting workers’ well-being [24, 92].

Workers who experienced bullying were more prone to rage and irritation, which have undesired consequences to the worker and the organisation. Thus, managers need to pay attention to these signals and act quickly to relieve workers of distressing feelings. Managers need to give workers channels to vent their rage since doing so would make them feel better [100]. Organisations could, for instance, offer victims psychological counselling services and listen to their complaints. Understanding the implicit expectations from fair treatment of workers may also help managers to manage and deliver on the expectations of employees, which in turn, helps prevent violations and other adverse outcomes. Perhaps, fostering a bottom-up approach to communication allows workers to report excessive job demands and low job resources and enables workers to talk about workplace abuse and hostility [35, 100–105]. Also, managers need to create a safe and decent psychosocial work environment that may lower the risk of workplace bullying and can successfully prevent the events leading to an escalation of vices and eventually increase productivity and organisational image [106–110].

### Recommendations for future research

Reviewed studies exploring PSC were mainly conducted in Australia, Malaysia and Canada, and not much research attention was given in Africa and South America. Also, existing PSC literature concentrates on occupational groups such healthcare workers, education workers, police and workers in the banking sector. Hence, studies from developing nations and other worker groups such as agricultural workers, road transport workers, rescue workers and military officers are needed. Moreover, the direct effect of PSC on some psychosocial work factors such as lone working, shift workers and those working extended hours may need more exploration. Furthermore, more studies are required to tease out the conditions under which the strength of PSC matters in the work context [12]. In addition, qualitative designs are needed to understand PSC through shared and individual experiences, working conditions and the psychological health of workers. More quality studies that adjust for confounding variables may be essential in understanding the independent effect of PSC on psychosocial work factors and stress symptoms. Finally, understanding PSC through the experiences of minority workers such as refugees, child workers, pregnant workers, and workers in the informal sectors might help improve the working conditions of vulnerable workers.

### Limitations in this review

About 63% of the included studies are cross-sectional surveys whose findings might be affected by response bias since they mostly rely on self-report measures. This situation may affect the generalisation of findings in this review. Also, the literature search was restricted to only peer-reviewed articles and papers published in English. This situation may affect the number of included studies and the depth of information presented in this review. Including only papers that explored PSC using PSC-12, PSC-8 and PSC-4 as measures may reduce the number of included studies which also affect the depth of information provided in this review. However, the authors pulled 93 studies from 45 countries globally, which may help understand PSC’s importance in creating a safe and healthy work environment for workers.

## Conclusion

Organisational PSC is an essential upstream job resource that directly affects psychosocial work factors, including job demands, job insecurity, effort-reward imbalance, work-family conflict, job resources, job control and quality leadership. In addition, PSC directly affects social relations at work, including workplace abuse, violence, discrimination and harassment. Moreover, PSC directly affects health, safety, and performance outcomes. Besides, PSC moderates the effect of working conditions on workers’ health, safety and performance across different occupational groups and settings. Therefore, designing quality jobs, prioritising the well-being of workers and fostering bottom-up communication through robust organisation policies, practices, and procedures may help create a high workplce PSC for healthy and decent work for all workers, for productivity and organisational integrity.

## Supplementary Information

**Additional file 1:** **Table S1.** Dataextracted from reviewed studies.

## Data Availability

All data generated or analysed during this study are included in this article and its supplementary information files (Table S1).
