# Supplementary material for: Influence of psychosocial safety climate on occupational health and safety: a scoping review
Source: BMC Public Health. 2023 Jul 13;23:1344. doi: 10.1186/s12889-023-16246-x (PMC10339512; doi:10.1186/s12889-023-16246-x)
Supplement: Supplementary file 1 — Additional file 1: Table S1. Dataextracted from reviewed studies. [file 12889_2023_16246_MOESM1_ESM.docx]

**Table S1: Data extracted from reviewed studies**

| **Authors/ country** | **Year of publication** | **Purpose of study** | **Design** | **Population** | **Sample size** | **PSC measure** | **Outcomes** | | |
| --- | --- | --- | --- | --- | --- | --- | --- | --- | --- |
|  |  |  |  |  |  |  | **Working conditions** | **Health and safety** | **Performance** |
| [13]  Ghana | 2018 | To examine the effect of PSC on Health and safety. | Cross-sectional survey | Fuel attendants | 876 | PSC-12 | Job demands (-s) and Job resources (+s) | Health and safety (+s). |  |
| [15]  Australia | 2022 | To explore the role of PSC in explain job demands and work-family conflict. | Cross-sectional survey | University workers | 2,191 | PSC-12 | Home digital job demands and work family conflict (-s). |  |  |
| [16]  Australia & Malaysia | 2012 | To examine the influence of PSC on job demands and psychological health. | Cross-sectional survey | Healthcare workers | 306 | PSC-12 | Job demands and psychological demands (-s). | Psychological well-being (+s). |  |
| [17]  Ghana | 2020 | To investigate the mediating role of PSC on the influence of job demands on health. | Cross-sectional survey | Fuel station attendants | 876 | PSC-12 | Job demands (-s). PSC mediates the relationship between job demands and health. | Health (+s). |  |
| [18]  Australia | 2010 | To validate the PSC-12 | Cross-sectional survey | General working population | 398 | PSC-12 | Job demands (-s) and Job resources (+s). | Health (+s) | Work engagement and job satisfaction (+s). |
| [19]  Malaysia | 2011 | To examine the influence of PSC on job demands and resources. | Cross-sectional survey | General working population. | 269 | PSC-12 | Job demands (-s) and Job resources (+s). |  |  |
| [20]  Malaysia | 2011 | To examine PSC is a precursor of job demands and resources. | Cross-sectional survey | General working population. | 291 | PSC-12 | Job demands (-s) and job resources (+s). | Burnout (-s) | Productivity (+s) |
| [21]  Malaysia | 2022 | To investigate the role of PSC and its association to work-related outcomes. | Cross-sectional survey | Oil and gas workers | 392 | PSC-12 | Cognitive demands (NS) and possibility for development (+S). |  |  |
| [22]  Iran & Australia | 2016 | To explore PSC in different cultural perspectives in Asia and Australia | Cross-sectional survey | Health workers | 496 | PSC-12 | PSC is viewed as a group phenomenon cross-culturally. Psychological demands (-s); Job resources, skill discretion and decision authority (+s) | Emotional exhaustion (-s). | Work engagement (+s). |
| [23]  Iran | 2019 | To examine the influence of PSC on mental health. | Cross-sectional survey | Healthcare workers | 247 | PSC-12 | Psychological and emotional demands (-s); organisational justice, supervisor support and organisational rewards (+s) | Emotional exhaustion and psychological distress (-s). | Work engagement (+s). |
| [24]  Canada | 2021 | The influence of PSC on working conditions and performance outcomes. | Longitudinal study | Teleworkers | 275 | PSC-4 | Psychological demands (-s). |  | Presenteeism remained unchanged while PSC increased over time. |
| [25]  Malaysia | 2014 | To explore the impact of PSC on job design and psychological outcomes. | Longitudinal study | Private sector workers | 370 | PSC-12 | Emotional demands (-s) | Emotional exhaustion (-s) |  |
| [26]  Australia. | 2010 | To assess PSC as a precursor to conducive work environment, psychological health and engagement. | Longitudinal study | Education workers | 288 | PSC-4 | Work pressure and emotional demands (-s). Skill discretion (+s). | Psychological distress and emotional exhaustion (-s). PSC moderated the effected of emotional demands on emotional exhaustion. | Work engagement (+s). |
| [27]  Australia | 2012 | To test the moderating role of PSC. | Longitudinal study | Police officers | 674 | PSC-12 | The interaction between demands and resources in predicting distress was moderated by PSC | Distress |  |
| [28]  Sweden | 2020 | To determine the benchmark for PSC for risk assessment. | Cross-sectional survey | General working population | 5,913 | PSC-4 | Quantitative demands (-s) and quality of leadership (+s). | Stress and burnout (-s). | Job commitment, engagement and satisfaction (+s). |
| [29]  Canada | 2022 | To test the moderating effect of PSC on the effect of work stress on presenteeism. | Longitudinal | Nurses | 800 | PSC-4 | Work intensification (-s). |  | Presenteeism. PSC moderates the effect of work intensification in presenteeism overtime. |
| [30]  Australia | 2015 | To examine PSC as precursor to psychosocial work factors. | Longitudinal study | General working population. | 1,095 | PSC-12 | Harassment, violence, bullying and work pressure (-s). |  |  |
| [31]  Australia | 2018 | To understand how PSC manifests among healthcare frontliners. | Mixed-method study | Healthcare frontliners | 27 | PSC-12 | PSC is applicable to group expectation and communication. PSC is capable of balancing conflicting pressures at work. |  | PSC help in improving quality of patient care. |
| [32]  Australia | 2021 | To assess the association between PSC and degression. | Longitudinal study | Full time employees | 2,023 | PSC-12 | PSC was not associated with long-working hours. | New major depressive symptoms (-s). The inverse relationship between PSC and depressive symptoms was stronger for females than males. |  |
| [33]  Malaysia | 2021 | To determine the predictors of job performance. | Cross-sectional survey | Hotel workers | 107 | PSC-12 | Hindrance demands (-S) |  | Job performance (+S). |
| [34]  Malaysia | 2021 | To examine the relationship between PSC and burnout. | Cross-sectional survey s | Hotel workers | 313 | PSC-12 | Challenge demands and hindrance demands (-S). | Burnout (-s) |  |
| [35]  Malaysia | 2021 | To explore PSC as a predictor of burnout. | Cross-sectional survey | Teachers | 413 | PSC-12 | Hindrance demands (-S) | Burnout (-S) |  |
| [36]  Malaysia | 2020 | To explore the linkage between PSC and burnout | review | Full time university academics |  |  | PSC determines working conditions, burnout and working conditions. |  |  |
| [37]  Malaysia | 2014 | To determine PSC as determinants of psychosocial working conditions and work outcomes. | Cross-sectional survey | Police | 909 | PSC-12 | Hindrance demands (-s) and Challenge demands (ns). |  |  |
| [38]  Italy | 2022 | To explore the influence of PSC on work engagement and psychological distress. | Cross-sectional survey | Frontline healthcare workers | 606 | PSC-12 | Working compulsively (-s). |  | Work engagement (+s) |
| [39]  Netherlands | 2015 | The impact of PSC on safety behaviour. | Cross-sectional survey | Healthcare workers | 6,230 | PSC-12 | Job demands (work family conflict and job insecurity) and job resources (co-workers support). |  | Safety behaviour (+s). PSC buffers the negative impact of work-family conflict and job insecurity on safety behaviours. PSC strengthens the impact of job resources on safety behaviour. |
| [40]  Canada | 2019 | To examine the influence of PSC on fatigue and exhaustion. | Cross-sectional survey | Nurses | 562 | PSC-12 |  | Physical fatigue, cognitive weariness and emotional exhaustion mediate the relationship between PSC and safety workaround. | Workaround (-s). |
| [41]  France | 2018 | To assess the impact of PSC in preventing ill-being. | Cross-sectional survey | Nurses | 269 | PSC-12 | Work-family conflict (-s) |  | Turnover intentions and need thwarting (-s) |
| [42]  Australia | 2016 | To examine PSC as an of effort-reward imbalance model extension. | Cross-sectional survey | General working population | 850 | PSC-12 | Effort-reward imbalance (-s) | Psychological distress and depression (-s). |  |
| [43]  Malaysia | 2017 | To explore the impact of PSC on team performance. | Cross-sectional survey | Workers in private companies | 412 | PSC-12 | Job resources (+s); Performance feedback and role clarity mediated the relationship between PSC and job engagement. |  | Job engagement (+s). |
| [44]  Malaysia | 2022 | To explore the effect of PSC on work investment and psychological health. | Longitudinal study | Polie officers | 392 | PSC-12 | Job resources (+s). PSC buffered the effect pf job resources on psychological distress and workaholism. |  | Work engagement and Workaholism (+s) |
| [45]  Malaysia | 2022 | To explore the effect of PSC on work engagement and commitment. | Cross-sectional survey | Lecturers | 484 | PSC-12 | Job resources (+s). Job resources mediated the relationship between PSC and work engagement. |  | Work engagement (NS) and Organisation commitment (+s). |
| [46]  Malaysia | 2019 | To test the role of PSC as a contextual factor in explaining emotional and cognitive resources. | Cross-sectional survey | Faculty members and college students | 350 | PSC-12 | Job resources (+s) for faculty members and emotional resources (+s) for students. |  |  |
| [47]  Australia | 2012 | To assess PSC ad a determinant of work characteristics and psychological distress. | Longitudinal study | Nurses | 365 | PSC-4 | Workload (-s), emotional demands (-s) control (+s) and supervisor support (+s). | Psychological strain (-s). |  |
| [48]  China | 2020 | To explore predictors of PSC in engineering construction projects | Cross-sectional survey | Construction workers | 624 | PSC-12 | Subjective initiatives (+s) | Improved mental well-being (self-worth) (+s) | Funding (+s) |
| [49]  Australia | 2010 | To examine the role of PSC in managing stress. | Longitudinal study | School staff | 288 | 8-item PSC | Decision influence (+s). | Psychological distress and emotional exhaustion |  |
| [50]  Malaysia | 2017 | To examine the impact of PSC on job resources and work engagement. | Cross-sectional survey | Teachers | 109 | PSC-12 | Managerial support (+s) |  |  |
| [51]  Pakistan | 2021 | To assess the impact of PSC on organisational citizenship behaviour. | Cross-sectional survey | Female  Nurses | 214 | PSC-12 | Perceived organisational support (+s). |  | Organisational citizenship behaviour (+s) |
| [52]  Australia | 2011 | To assess PSC as a lead indicator of psychosocial work factors. | Cross-sectional survey | General working population | 1,134 | PSC-12 | Workplace harassment and bullying (-s). Rewards (+s). | PSC moderated the effect of bullying and workplace harassment on psychological health problem. |  |
| [53]  Portugal | 2022 | To examine the impact of PSC on work engagements and affective commitment. | Cross-sectional survey | Hotel workers | 217 | PSC-12 | Organisational justice (+s) |  | Affective commitment (+s). |
| [54]  Malaysia | 2021 | To examine the mediating role of PSC | Quasi-experiment | Oil and gas workers | 303 | PSC-12 | Health-centric leadership (+s). | Psychological distress (-s). PSC mediates the association between health-centric leadership styles and psychological health. |  |
| [55]  Finland | 2022 | To understand the impact of PSC on psychological safety. | Qualitative | Remote university staff | 26 |  | Psychosocial working conditions and good leadership. |  | Psychological safety |
| [56]  Australia | 2022 | To explore ethe impact of PSC on employees’ psychological coping resources. | Cross-sectional survey | Healthcare workers | 163 | PSC-12 | Psychological capital (+s) | Well-being (+s) | Innovative behaviour (+s) |
| [57]  Australia | 2010 | The influence of workplace bullying and PTSD. | Longitudinal study | Police officers | 934 | 8-item PSC | Workplace bullying (-s) | PTSD. PSC moderated the effect of workplace bullying on PTSD. |  |
| [58]  Vietnam | 2017 | To assess the role of PSC in a hostile workplace environment. | Cross-sectional survey | General working population | 274 | PSC-12 | Workplace bullying (-s). | Well-being (+s) | Work engagement (+s). PSC moderated the effect of workplace bullying on work engagement. |
| [59]  Australia | 2017 | To assess the role of PSC in reducing workplace bullying and psychological health problem. | Longitudinal study | General population | 1,062 | PSC-12 | Workplace bullying (-s) | Psychological health (+s) |  |
| [60]  USA | 2021 | To examine the distinction between PSC and the Stigma in reporting stress. | Cross-sectional survey | General working population | 680 | PSC-12 | Bullying (-s). PSC played a moderating role in attenuating the effect of Stigma on bullying and burnout. | Burnout (-s). |  |
| [61]  Malaysia | 2016 | To examine the role of PSC in coping with workplace abuse. | Qualitative | General working population. | 20 | PSC-12 | Employees tended to voice in high PSC contexts, which led to swift resolution of  Bullying. In low PSC, bullying issues remained unresolved and led to turnover intentions and neglection. |  | Turnover intentions |
| [62]  Spain | 2021 | To understand if PSC may explain emotional exhaustion. | cross-sectional survey | General working population. | 4,982 | PSC-4 | Bullying (-s) | Emotional exhaustion (-S). |  |
| [63]  China | 2019 | To assess the influence of PSC on workplace violence and self-rated health. | Cross-sectional survey | Nurses | 1,690 | PSC-12 | Workplace violence (-s) | Poor self-rated health (-s). |  |
| [64]  Australia | 2021 | To examine the role of PSC against workplace abuse and psychological distress. | Cross-sectional survey | Refuges workers | 117 | PSC-4 | Workplace abuse and discrimination (-s). | Psychological distress (-s) |  |
| [65]  Canada | 2022 | To examine the influence of PSC on managerial quality. | Cross-sectional survey | Managers | 105 | PSC-12 |  | Burnout (-s) | Managerial quality (+s). |
| [66]  Australia | 2015 | To determine benchmark for PSC and the impact of PSC on reducing mental health issues at work. | Longitudinal study | General working population | 4,221 | PSC-12 |  | Job strain and depression (-s) |  |
| [67]  USA | 2021 | To examine the influence of PSC on physical and psychological impact of perfectionism. | Longitudinal study | Attorneys | 176 | PSC-12 |  | Physical and psychological distress (-s) |  |
| [68]  Australia | 2017 | To examine the role of PSC in explaining emotional exhaustion and work injuries. | Longitudinal study | Healthcare workers | 214 | PSC-12 |  | Emotional exhaustion and Injuries (-s). |  |
| [69]  Canada | 2018 | To test the direct and indirect effect of PSC on work-family conflict. | Cross-sectional survey | Nurses | 562 | PSC-12 | WFC and FWC (-s). |  |  |
| [70]  Australia | 2014 | To test the moderating effect of PSC on the effect of job demands on recovery fatigue and work engagement. | Longitudinal study | School teachers | 61 | PSC-12 (used 11 items). |  | Fatigue (-s). PSC moderated the effect of job demands and recovery on fatigue. | Work engagement (+s). PSC moderated the effect of job demands and recovery on work engagement. |
| [71]  Australia | 2019 | To examine the dynamic interplay of physical safety climate and PSC | Longitudinal study | Healthcare workers | 463 | PSC-12 |  | Accidents (-s) | Absence (-s) and patient safety (+s). |
| [72]  Australia | 2018 | To explore the impact of PSC on circulatory diseases. | Longitudinal study | General working population. | 1,223 | PSC-12 |  | Circulatory diseases (low PSC create 59% chances of developing new CD) |  |
| [73]  Netherlands | 2017 | To examine the association between PSC and stress | Cross-sectional survey | Healthcare workers | 277 | PSC-12 |  | Stress (-s) |  |
| [74]  Australia | 2018 | To examine PSC as a risk factor for depression. | Longitudinal study | General working population | 1,905 | PSC-12 |  | Depression (-s) |  |
| [75]  Austria; Belgium; Bulgaria; Cyprus; Czech Republic; Denmark; Estonia; Finland; France; Germany; Greece;  Hungary; Ireland; Italy;  Latvia; Lithuania; Luxembourg; Malta;  Netherlands; Poland; Portugal; Romania; Slovakia; Slovenia; Spain;  Sweden; UK; Switzerland; Croatia; Turkey and Norway | 2013 | To determine PSC as a determinant of workers health and productivity. | Cross-sectional survey | OHS managers | 28,649 |  |  | Health (+s) | Productivity (+s). |
| [76]  Iran | 2022 | To assess the role of PSC as an enhancer of hope and resilience. | Cross-sectional survey | Healthcare workers | 623 | PSC-12 | PSC moderated the effect of supportive leadership on Personal hope. | Personal resilience and hope (+s) |  |
| [77]  Malaysia | 2018 | To determine the risk factors for PSC | Quasi-experiment | Police officers | 105 | PSC-12 | Team psychological safety and physical safety climate (+s). The introduction of occupational safety climate increased PSC. |  |  |
| [78]  China | 2022 | To explore PSC impact on turnover intentions and job satisfaction. | Cross-sectional survey | Construction workers | 480 | PSC-12 |  |  | Job satisfaction and intentions to stay (+S). |
| [79]  Sweden | 2019 | To examine the role of PSC in explaining job engagement and commitment. | Cross-sectional survey | Social workers | 725 | PSC-4 |  |  | Job satisfaction (+s). |
| [80]  Ghana | 2020 | To explore the moderating role of PSC on the effect of psychosocial hazards and work engagement. | Cross-sectional survey | Bankers | 543 | PSC-12 |  |  | Work engagement (+S). PSC moderated the effect of workplace bullying on work engagement. |
| [81]  Malaysia | 2019 | To examine the influence of PSC on proactive motivation. | Longitudinal study | Private company employees | 134 | PSC-12 |  |  | Personal development, work engagement and personal initiative (+s). |
| [82]  United Arab Emirate | 2022 | To explore the moderating role of PSC. | Longitudinal study | Health workers | 121 | PSC-4 | PSC moderated the relationship between Job demands and burnout; and between social support and work engagement. | Burnout (NS) | Work engagement (NS). |
| [83]  Netherlands | 2016 | To examine the effect of organisational safety climate and performance outcomes. | Cross-sectional survey | Healthcare workers | 8,761 | PSC-12 |  |  | Absenteeism and presenteeism (-S). |
| [84]  Australia | 2013 | To examine PSC in the aged care facilities. | Cross-sectional survey | Aged home workers | Not stated | PSC-12 |  | Management strain (-s) | High morale, sustained profits and reciprocal behaviour (+s). absenteeism and claims (-s). |
| [85]  China | 2020 | To assess the impact of PSC on reducing ill-health presenteeism. | Longitudinal study | Healthcare workers | 386 | PSC-12 |  |  | Ill-heath presenteeism (-s). |
| [86]  China | 2020 | To explore the association between PSC and unsafe work behaviours. | Cross-sectional survey | Miners | 862 | PSC-12 |  |  | Unsafe working behaviour (-S). |
| [87]  Nigeria | 2021 | To examine the effect of PSC on workplace safety behaviours. | Cross-sectional survey | Bankers | 155 | PSC-12 |  |  | Workplace safety behaviours (+S). |
| [88]  Malaysia | 2022 | To explore ethe impact of PSC on safety behaviours. | Cross-sectional survey | Oil and gas workers | 190 | PSC-12 |  | Psychological distress (-s) | Safety compliance and participation (+s). |
| [89]  Iran | 2023 | To examine if PSC improves behaviour outcomes among service workers. | Cross-sectional survey | Bankers | 50 | PSC-12 |  |  | Positive service behaviour (+S). |
| [90]  Australia | 2022 | To understand the influence of PSC on creative decision making. | Mixed method study | Engineers | 25 | PSC-12 |  |  | Creative problem solving (+s). |
| [91]  Canada, France & Germany | 2022 | To examine the mediating role of PSC on the relationship between high performance work practices and performance. | Cross-sectional survey | Flight attendants | 1,664 | PSC-12 | Ability enhancing, motivating enhancing and opportunity enhancing (+s). |  | Service recovery performance (+s). |
| [92]  Canada | 2018 | To examine the effect of PSC on managerial quality. | Cross-sectional survey | Managers | 192 | PSC-12 | Job control mediated the relationship between PSC and Managerial quality. Workload, social support and relationship quality with subordinates did not mediate the relationship between PSC and managerial quality. |  | Managerial quality (+S) |
| [93]  India | 2018 | To examine the moderating role of PSC on the effect of bullying on psychological contract violation. | Cross-sectional survey | Managerial workers | 835 | PSC-12 |  |  | Psychological contract violation (PSC moderated the relationship but showed an inverse results) |
| [94]  Australia | 2013 | To test the moderating role of PSC on the effect of job demands and depression. | Cross-sectional survey | General working population | 2,343 | PSC-12 | PSC moderated the effect of job demands on depression and the effect of depression on positive organisational behaviours. |  |  |
| [95]  Malaysia | 2018 | Testing PSC as a moderator of moderators. | Longitudinal study | Healthcare workers | 429 | PSC-12 | PSC was a strong moderator of emotional demand on psychological health problems (emotional exhaustion and somatic symptoms). |  |  |
| [96]  Australia | 2018 | To explore the impact of PSC on mindfulness. | Experiment | Education, healthcare and finance. | 57 | PSC-12 | As PSC was increased, job control was positively associated with everyday mindfulness at work. |  |  |
| [97]  Australia | 2017 | To examine the impact of perceived PSC over jurisdictions. | Longitudinal study | General working population | 3,383 | PSC-12 | PSC levels significantly decreased in the non-harmonised jurisdiction over time. Thus, without harmonisation, PSC level is reduced. |  |  |
| [98]  Malaysia | 2022 | To examine the role of PSC in enhancing psychological detachment and relaxation. | Cross-sectional survey | Teachers | 178 | PSC-12 | The interaction role of PSC*psychological detachment and PSC*relaxation moderated the relationship between daily job demands and daily emotional  exhaustion |  |  |
| [102]  Indonesia | 2021 | The influence of PSC on work engagement. | Cross-sectional survey | SMEs employees | 164 | PSC-4 |  |  | Work engagement (+s). |
| [103]  Malaysia | 2022 | To assess the influence of PSC on burnout | Cross-sectional survey | University academicians | 686 | PSC-12 |  | Burnout (-s). |  |
| [104]  Iran | 2022 | The influence of PSC on customers engagement. | Cross-sectional survey | Insurance company managers, service employees and customers | 1,129 | PSC-12 |  |  | Adaptive and proactive service behaviours (+s). |
| [105]  Australia | 2016 | To understand the PSC of pre-school environment. | Qualitative | Pre-school teachers. | 16 |  | Pre-school had policies and procedures that value teachers’ well-being and psychological safety. |  | Teachers experience of PSC influence their beliefs and behaviours in the classroom. |
| [106]  Malaysia | 2021 | Review literature on use of PSC to create healthy working environment. | Review | Education |  |  | Healthy job designs, enhancing job demands and diminishing hinderance demands. | Minimise burnout |  |
| [107]  Iran | 2020 | The impact of government policies on PSC | Review | General working population |  |  | Poor policies on quarantine and communication had negative impact on PSC. |  |  |
| [108]  Norway | 2023 | To examine the moderating role of PSC | Cross-sectional survey | General working population | 15,524 | PSC-8 | Workplace bullying (-s).  PSC moderated the association between role conflict and workplace bullying. PSC further moderated the association between role ambiguity and workplace bullying. |  |  |
| [109]  Japan | 2023 | To examine PSC as predictors of psychological distress and work engagement | Cross-sectional survey | General Working Population | 2,200 | PSC-12 |  | Psychological distress (-s) | Work engagement (+s) |

(-s) = significant negative association with PSC; (+s) = significant positive association with PSC; (NS) = No significant association with PSC.
